# Enhancing Protein Content in Wild-Type *Saccharomyces cerevisiae* via Random Mutagenesis and Optimized Fermentation Conditions

**DOI:** 10.4014/jmb.2405.05027

**Published:** 2024-07-31

**Authors:** Sang-Hun Do, Tae-Gi Lee, Sun-Ki Kim

**Affiliations:** 1 Department of Food Science and Biotechnology, Chung-Ang University, Anseong 17546, Republic of Korea; 2 GreenTech-based Food Safety Research Group, BK21 Four, Chung-Ang University, Anseong 17546, Republic of Korea

**Keywords:** *Saccharomyces cerevisiae*, single-cell protein, random mutagenesis, molasses, fed-batch fermentation

## Abstract

Single-cell protein (SCP) derived from microorganisms is widely recognized as a viable alternative protein source for the future. Nevertheless, the commercialization of yeast-based SCP is hampered by its relatively low protein content. Therefore, this study aimed to enhance the protein content of *Saccharomyces cerevisiae* via random mutagenesis. To achieve this, *S. cerevisiae* KCCM 51811, which exhibited the highest protein concentration among 20 edible *S. cerevisiae* strains, was selected as a chassis strain. Subsequently, a KCCM 51811 mutant library was constructed (through UV irradiation) and screened to isolate mutants exhibiting high protein content and/or concentration. Among the 174 mutant strains studied, the #126 mutant exhibited a remarkable 43% and 36% higher protein content and concentration, respectively, compared to the parental strain. Finally, the #126 mutant was cultured in a fed-batch system using molasses and corn-steep liquor, resulting in a protein concentration of 21.6 g/l in 100 h, which was 18% higher than that produced by the parental strain. These findings underscore the potential of our approach for the cost-effective production of food-grade SCP.

## Introduction

The demand for meat protein is anticipated to surge by 72% between 2013 and 2050 owing to the significant increase in meat consumption [1]. However, conventional meat production systems raise several environmental issues, including exacerbated soil erosion, excessive water consumption, and greenhouse gas emissions [2]. Notably, the current animal-mediated food production system accounts for one-third of the global anthropogenic greenhouse gas emissions [3]. Therefore, consumer demand for alternative proteins is growing, with the global market projected to reach USD 29 billion in 2035 [4]. Among the various alternative protein sources, single-cell proteins derived from microbes have recently received considerable attention due to their high yields, rapid production rates, and minimal arable land requirements [5]. Notably, a recent study reported that replacing 20%of the global per capita ruminant meat consumption with SCP would not only offset future increases in global pasture area but also reduce annual deforestation and related CO_2_ emissions by half by 2050 [6].

Among the various candidate microbes capable of producing SCP, *Saccharomyces cerevisiae* was selected as the SCP producer in this study because it is generally recognized as safe (GRAS), has high nutritional value, and is widely employed in the food industry [7]. However, the protein content of yeast is usually 15–30% lower than that of bacteria [7]. Previously, we isolated a mutant of the *S. cerevisiae* D452-2 strain that produced high protein content [8]. However, it could not be readily used in the food industry because the background D452-2 strain is a genetically modified microorganism (GMM).

To circumvent this limitation and enhance the protein content in edible *S. cerevisiae* for food-grade SCP production, a wild-type *S. cerevisiae* with high protein content was selected and used for mutant library preparation through UV irradiation. Then, the mutant with a notably improved protein content compared to the parent strain was isolated and cultured using molasses and corn-steep liquor (CSL) in a fed-batch system. Using this method, the isolated mutant strain yielded a total protein concentration of 21.6 g/l.

## Materials and Methods

### *Saccharomyces cerevisiae* Strains

A laboratory *S. cerevisiae* strain, D452-2 [9], and 20 wild-type *S. cerevisiae* strains from the Korean Agricultural Culture Collection (KACC) and Korean Culture Center of Microorganisms (KCCM) were used in this study, as detailed in Table S1.

### Media and Culture Conditions

The *S. cerevisiae* strains were pre-cultured in 5 ml YP20D medium (10 g/l yeast extract, 20 g/l Bacto Peptone, and 20 g/l glucose) for 48 h at 30°C and 250 rpm. During batch fermentation, pre-cultured cells were harvested and inoculated into main cultures with an initial optical density at 600 nm (OD_600_) of 1.0. The main fermentation was performed in 100 ml YP50D medium (10 g/l yeast extract, 20 g/l Bacto Peptone, and 50 g/l glucose) for 36 h at 30°C and 250 rpm.

Fed-batch fermentation was performed in a 2.5 L bioreactor (Kobiotech Co., Republic of Korea) containing 1 L of a mixture of 4% (v/v) molasses (Konem Bio Co., Republic of Korea) and 2% (v/v) CSL (Shanghai Aladdin Bio-Chem Technology Co., China). Following the depletion of sugars present in 4% (v/v) molasses, a feeding solution consisting of 50% (v/v) molasses and 25% (v/v) CSL was fed at a rate ranging from 7.6 to 22.5 ml/h. The medium temperature, pH, air supply, and agitation speed were maintained at 30°C, pH 5.5, 2.0 vvm, and 1,000 rpm.

### Screening of Mutants with Improved Protein Content and Concentration

A mutant library was constructed through UV irradiation, following a previously reported method [8]. A total of 174 single colonies picked from the mutant library were cultivated in 200 μl of YP20D medium for 48 h at 30°C and 900 rpm. After harvesting cells via centrifugation, the cell pellets were resuspended in 100 μL of Y-PER Yeast Protein Extraction Reagent (Thermo Fisher Scientific, USA) and lysed following the manufacturer’s instructions. Protein concentrations (g protein/l) were determined using the kit (USA), with bovine serum albumin as the standard. Protein content (g protein/g cell × 100, %), was calculated by dividing the protein concentration by the dry cell weight (DCW) concentration.

### Analytical Methods

A spectrophotometer (OPTIZEN POP, Mecasys, Republic of Korea) was used to measure cell growth at OD_600_, and the DCW of the KCCM 51811 strain was calculated by multiplying the OD_600_ value by a conversion factor of 0.6 g/l/OD. Ethanol concentration was determined using an HPLC system (Ultimate 3000, Thermo Fisher Scientific) with a Rezex ROA-Organic Acid H+ column (Phenomenex, USA). The column was eluted with 5 mM H_2_SO_4_ at 60°C and a flow rate of 0.6 ml/min, and metabolites were detected using a reflective index detector.

To analyze total amino acid concentration, 2–3 mg of the protein from cell lysates was hydrolyzed with 30 ml of 6 N HCl for 48 h at 30°C. After centrifugation, the supernatant was diluted 10-fold with ultrapure water, and the pH was adjusted to 7. Subsequently, the amino acids were derivatized using 9-fluorenylmethoxycarbonyl chloride and o-phthalaldehyde and analyzed using an HPLC system (Ultimate 3000, Thermo Fisher Scientific) equipped with an Inno C18 column (YoungJin Biochrom Co., Republic of Korea), following the methods outlined in a previous study [8].

### Statistical Analysis

Statistical analyses were performed using IBM SPSS version 28 (IBM, USA). All data are presented as the mean± standard deviation. One-way analysis of variance was performed, and statistical significance was assessed using Tukey’s Honestly Significant Difference (HSD) tests, with the significance level set at *p* < 0.05.

## Results and Discussion

### Selection of a Wild-Type *Saccharomyces cerevisiae* with High Protein Concentration

**Fig. 1:**
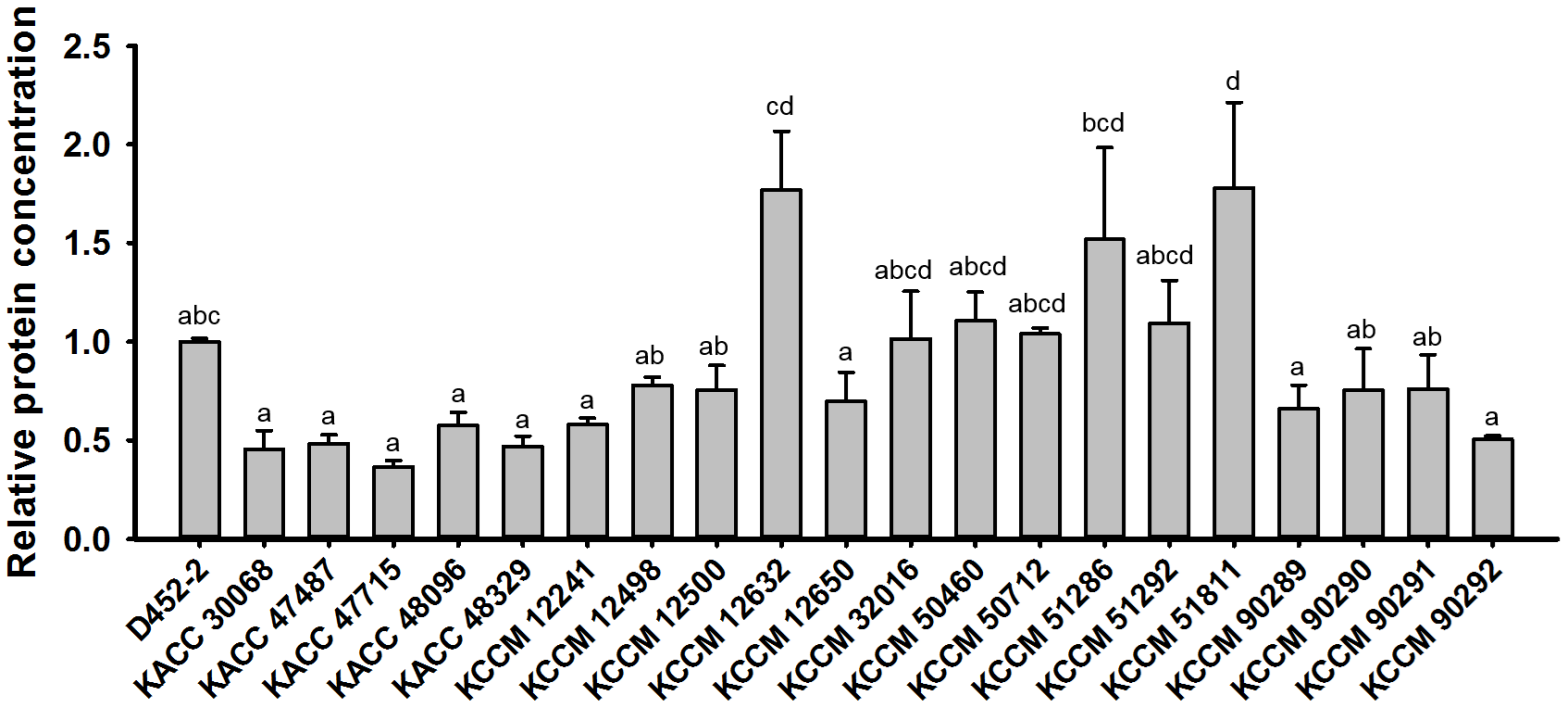
Comparison of protein concentrations in the *Saccharomyces cerevisiae* laboratory strain, D452-2, and 20 wild-type *S. cerevisiae* strains. The relative fold change was calculated based on the protein concentration of the sample strains relative to that of the D452-2 strain. The *S. cerevisiae* strains were cultured in 100 ml YP50D medium (10 g/l yeast extract, 20 g/l Bacto Peptone, and 50 g/l glucose) at 30°C and 250 rpm for 36 h. Results represent the mean of two experiments, with error bars indicating the standard deviation. Different letters represent significantly different means (Tukey HSD tests, *p* < 0.05).

To select the GRAS yeast strain capable of producing SCP at high concentrations, the protein concentrations of 20 edible *S. cerevisiae* strains (Table S1) were compared using the Bradford protein assay. While this method is not considered accurate for protein quantification [10], it facilitated an easy comparison of relative protein concentrations between strains. The protein concentrations of the candidate strains were precisely determined using an HPLC-based amino acid profiling method. Among the 20 wild-type *S. cerevisiae* strains studied, the KCCM 12632, KCCM 51286, and KCCM 51811 strains cultivated in YP50D medium exhibited significantly higher protein concentrations than the control laboratory strain, D452-2 (Fig. 1). The amino acid profiling of these three strains via HPLC revealed that the KCCM 12632 strain exhibited the highest total amino acid concentration at 6.2 g/l (Fig. S1). However, the KCCM 12632 strain was excluded from further consideration because its highly flocculent nature renders it unsuitable for industrial-scale mass fermentation. Among the remaining strains (KCCM 51286 and KCCM 51811), KCCM 51811 was selected as the background strain for SCP production because only this strain could facilitate further enhancement in protein concentration through random mutagenesis.

### Fermentation Using Molasses and CSL to Validate the Feasibility of Large-Scale SCP Production

**Fig. 2:**
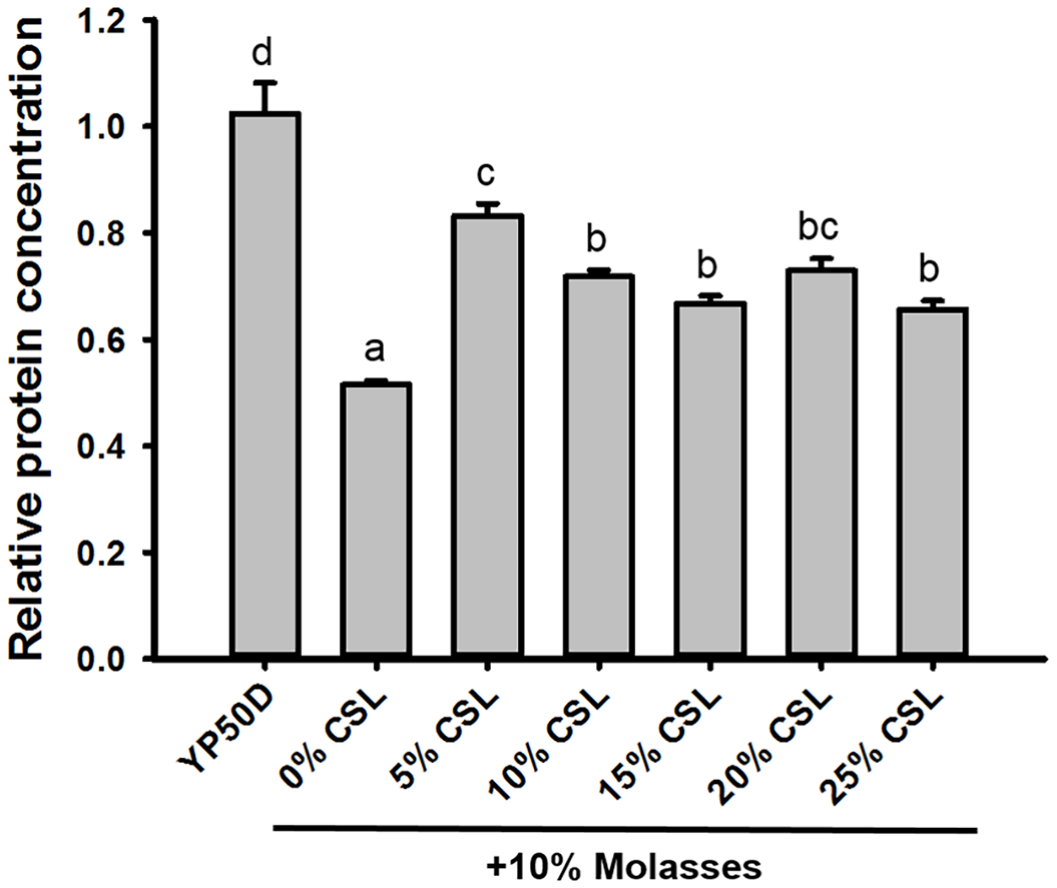
Comparison of protein concentrations produced by the *Saccharomyces cerevisiae* KCCM 51811 strain in YP50D medium and a mixture of 10% (v/v) molasses containing various concentrations (0, 5, 10, 15, 20, and 25% (v/v)) of corn steep liquor (CSL). The relative fold change was calculated based on the protein concentration in the molasses and CSL mixture relative to that in the YP50D medium (10 g/l yeast extract, 20 g/l Bacto Peptone, and 50 g/l glucose). The *S. cerevisiae* strains were cultured in various media at 30°C and 250 rpm for 36 h. Results represent the mean of *n* ≥ 2 experiments, with error bars indicating the standard deviation. Different letters represent significantly different means (Tukey HSD tests, *p* < 0.05).

The food industry frequently utilizes complex media composed of molasses and CSL, a byproduct of the sugar and corn wet milling industries, respectively, for cost-effective large-scale fermentation [11, 12]. CSL is one of the cheapest nitrogen sources [13], and molasses contain ~50% sugars (fructose, glucose, and sucrose), ~25%polysaccharides, vitamins, and nitrogen sources [11]. Accordingly, we compared the protein concentration produced by the KCCM 51811 strain cultivated in YP50D medium with that produced by the KCCM 51811 strain cultivated in a complex medium containing 10% (v/v) molasses and various concentrations (0, 5, 10, 15, 20, and 25% (v/v)) of CSL. To ensure a sugar concentration equivalent to the 50 g/l glucose concentration present in the YP50D medium, the concentration of molasses was maintained at 10% (v/v). As shown in Fig. 2, the KCCM 51811 strain exhibited a 48% lower protein concentration when cultured in the medium containing 10% (v/v) molasses and no CSL compared to when cultured in the YP50D medium, likely due to limited nitrogen availability. Furthermore, consistent with our expectations, the addition of 5% (v/v) CSL to the medium containing 10% (v/v) molasses (10M5C) significantly improved the protein concentration of the KCCM 51811 strain, achieving 83% of that produced in the YP50D medium. The increased protein concentration observed in the KCCM 51811 strain cultivated in 10M5C medium is due to a higher maximum DCW, rather than an increase in protein content (data not shown). However, further supplementation with CSL did not increase the protein concentration produced by the KCCM 51811 strain (Fig. 2). Considering that molasses and CSL are much less expensive than the yeast extract, peptone, and glucose used in the YP50D medium, we concluded that the 10M5C medium represents a suitable option for the cost-effective production of SCP.

### Isolation of KCCM 51811-Derived Mutants for Efficient SCP Production

**Fig. 3:**
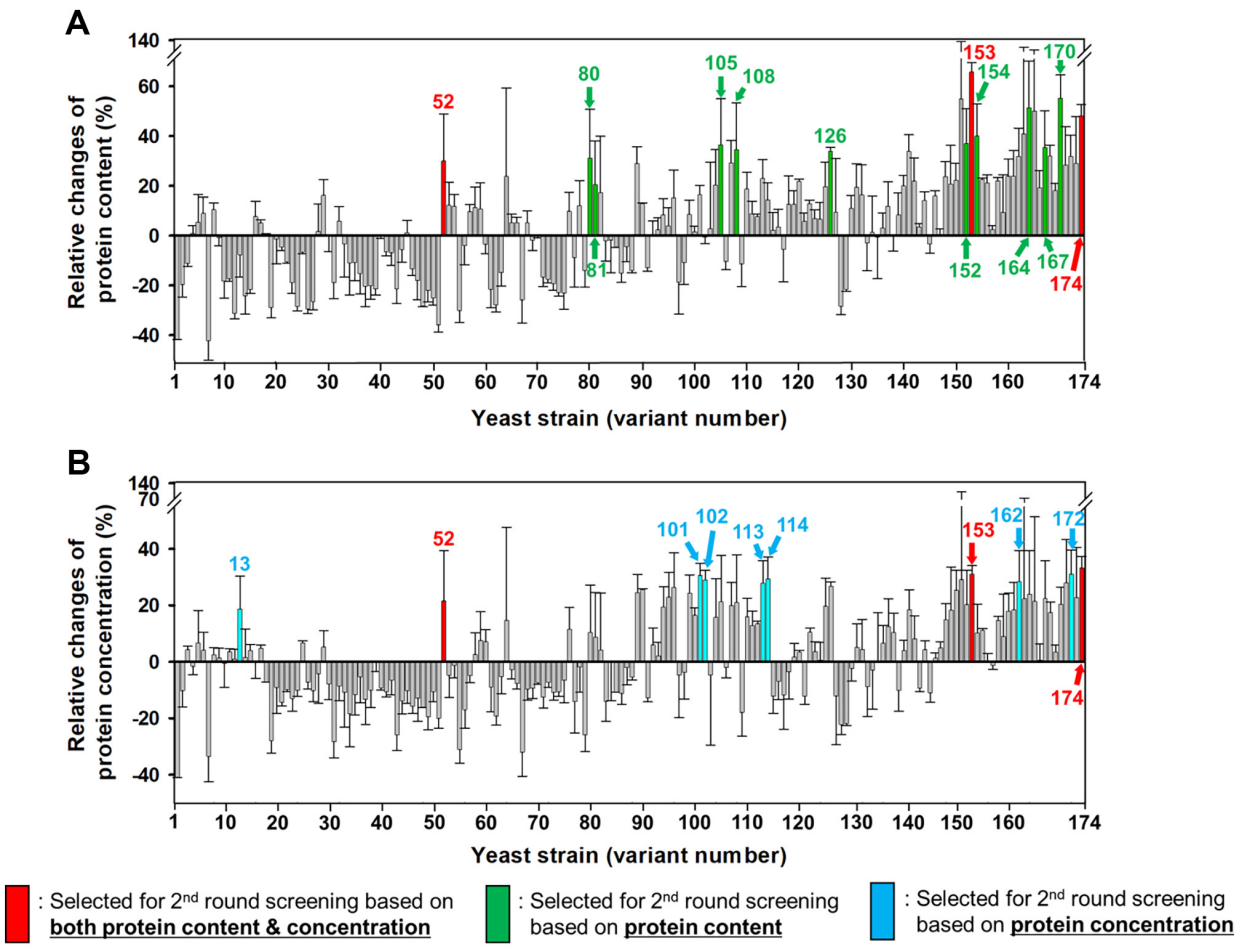
Comparison of protein contents (A) and concentrations (B) in the parental *Saccharomyces cerevisiae* KCCM 51811 and its mutant strains. The relative fold change was calculated based on the protein contents and concentrations of the sample strains relative to those of the KCCM 51811 strain. In total, 174 single colonies derived from the mutant library were cultured in 200 μl YP20D medium (10 g/l yeast extract, 20 g/l Bacto Peptone, and 20 g/l glucose) at 30°C and 250 rpm for 48 h. Results represent the mean of two experiments, with error bars indicating the standard deviation.

Although the protein content of the KCCM 51811 strain is already high, greater protein content is more advantageous for commercial SCP production. To achieve this, a KCCM 51811 mutant library with a 2% survival rate upon UV exposure was generated, consistent with previous studies indicating that the optimal UV exposure time for constructing a reliable mutant library with high mutation density is when the number of viable cells decreased by 97–98% [8, 14]. A total of 174 single colonies were randomly selected from the mutant library and cultivated in 96-well plates for 48 h to isolate mutants with improved protein content and/or concentration. Fermentation at a small scale of 200 μl for cultivating a large number of mutants in parallel using the 10M5C medium posed challenges due to precipitate formation. Therefore, we used the YP medium for screening mutants with improved SCP production, and a medium containing molasses and CSL was selected for the mass production of SCP. Twenty out of the 174 mutants were selected based on the following criteria (Fig. 3): (1) higher protein content than the parental KCCM 51811 strain (#52, #80, #81, #105, #108, #126, #152, #153, #154, #164, #167, #170, and #174), (2) higher protein concentration than the parental KCCM 51811 strain (#13, #52, #101, #102, #113, #114, #153 #162, #172, and #174), and (3) higher protein content and concentration than the parental KCCM 51811 strain (#52, #153, and #174).

**Fig. 4:**
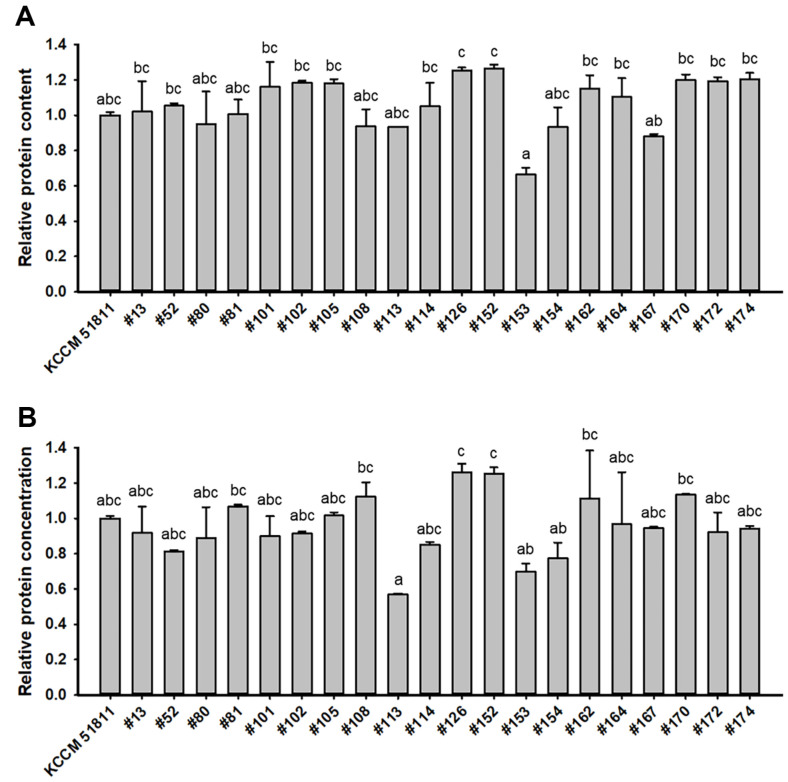
Comparison of protein contents (A) and concentrations (B) in the parental *Saccharomyces cerevisiae* KCCM 51811 and its twenty mutant strains from the first-round screening. The relative fold change was calculated based on the protein contents and concentrations of the sample strains relative to those of the KCCM 51811 strain. The *S. cerevisiae* strains were cultured in 100 ml YP50D medium (10 g/l yeast extract, 20 g/l Bacto Peptone, and 50 g/l glucose) at 30°C and 250 rpm for 36 h. Results represent the mean of *n* ≥ 2 experiments, with error bars indicating the standard deviation. Different letters represent significantly different means (Tukey HSD tests, *p* < 0.05).

**Fig. 5:**
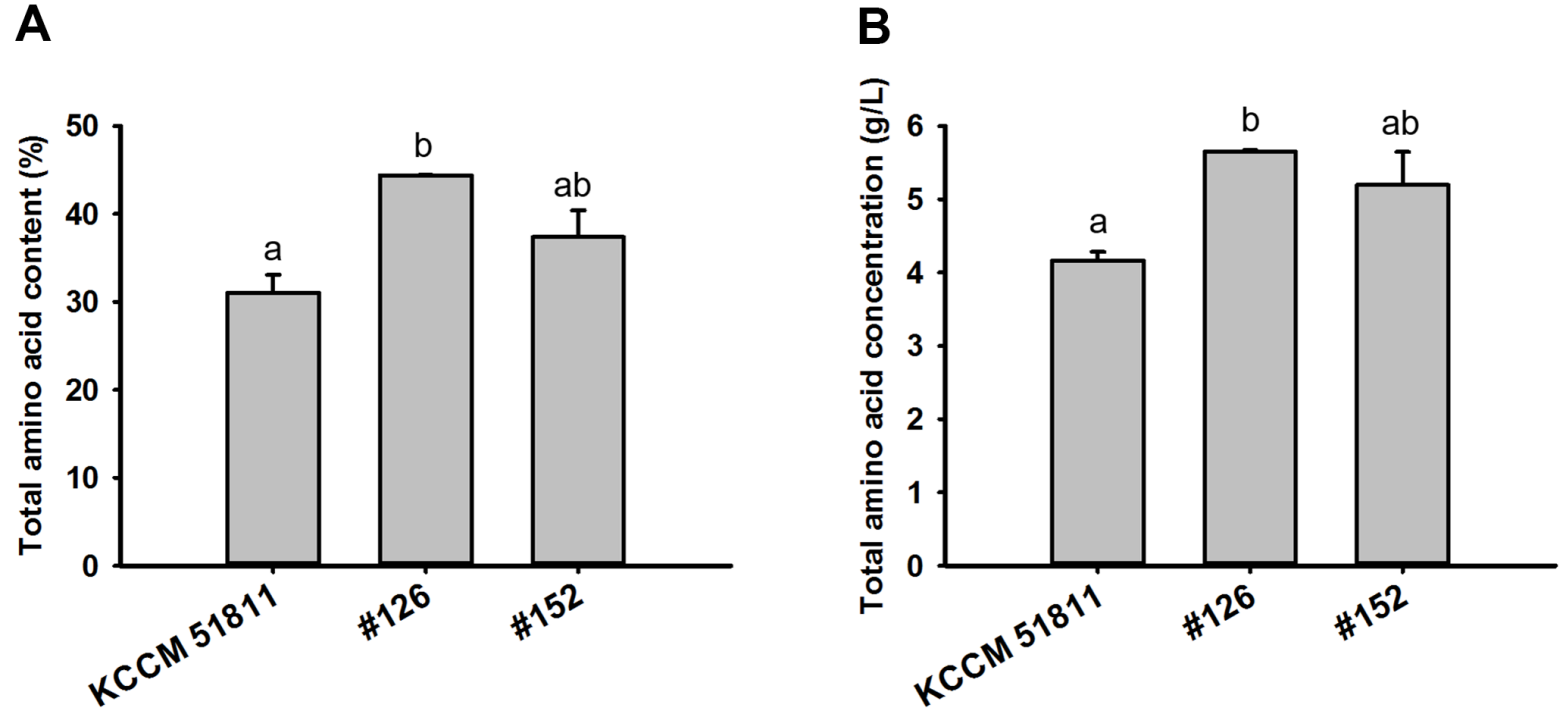
Comparison of total amino acid contents (A) and concentrations (B) in the parental *Saccharomyces cerevisiae* KCCM 51811 and its two mutant strains (#126 and #152). Total amino acid contents and concentrations were calculated through HPLC-based amino acid profiling. Results represent the mean of *n* ≥ 2 experiments, with error bars indicating the standard deviation. Different letters represent significantly different means (Tukey HSD tests, *p* < 0.05).

The 20 mutants selected through the 200 μl-scale screening were cultured in 100 ml YP50D medium for the second-round screening. However, the overall increase in protein content and/or concentration observed in the 100 ml-scale screening was lower than that observed in the 200 μL-scale fermentation (Fig. 4). This result is likely attributed to the differences in fermentation conditions, including aeration and agitation, which are strongly influenced by fermentation scale. Nevertheless, the #126 and #152 mutant strains still exhibited more than a 25%increase in protein content and concentration in the 100 ml-scale fermentation (Fig. 4). To more accurately compare SCP production capacity, protein contents and concentrations of the #126 and #152 mutants were measured using the HPLC-based amino acid profiling method. The #126 and #152 mutant strains exhibited higher amino acid contents and concentrations than the parental KCCM 51811 strain for all amino acid types measured (Tables 1 and S2). Specifically, the #126 mutant exhibited a 43% and 36% higher total amino acid content and concentration, respectively, whereas the #152 mutant exhibited a 20% and 25% higher total amino acid content and concentration, respectively, compared to the parental strain (Fig. 5). Considering that Angel (Angel Yeast Co., Ltd., China), a commercially available high-protein yeast, typically possesses a protein content of 38% [15], the #126 mutant, with a protein content of 44%, is considered sufficient for commercialization. Because the yeast culture conditions and the methods used to determine the protein content of Angel differ from those employed in the present study, the preceding values are for reference only. In our previous study, we successfully identified single nucleotide polymorphisms (SNPs) responsible for the desired phenotype using next-generation sequencing [8]. However, identifying the SNPs responsible for enhanced SCP production in the #126 mutant strain is a challenge because wild-type *S. cerevisiae* strains typically have complex polyploid genomes [16]. For instance, identifying SNPs in polyploids requires distinguishing between homeologous and allelic SNPs [17]. Additional experiments are currently underway for optimizing the sequencing, analysis, and filtering strategies for SNP identification in polyploids.

### SCP Production Using the #126 Mutant Strain in a Fed-Batch System with Molasses and CSL

**Fig. 6:**
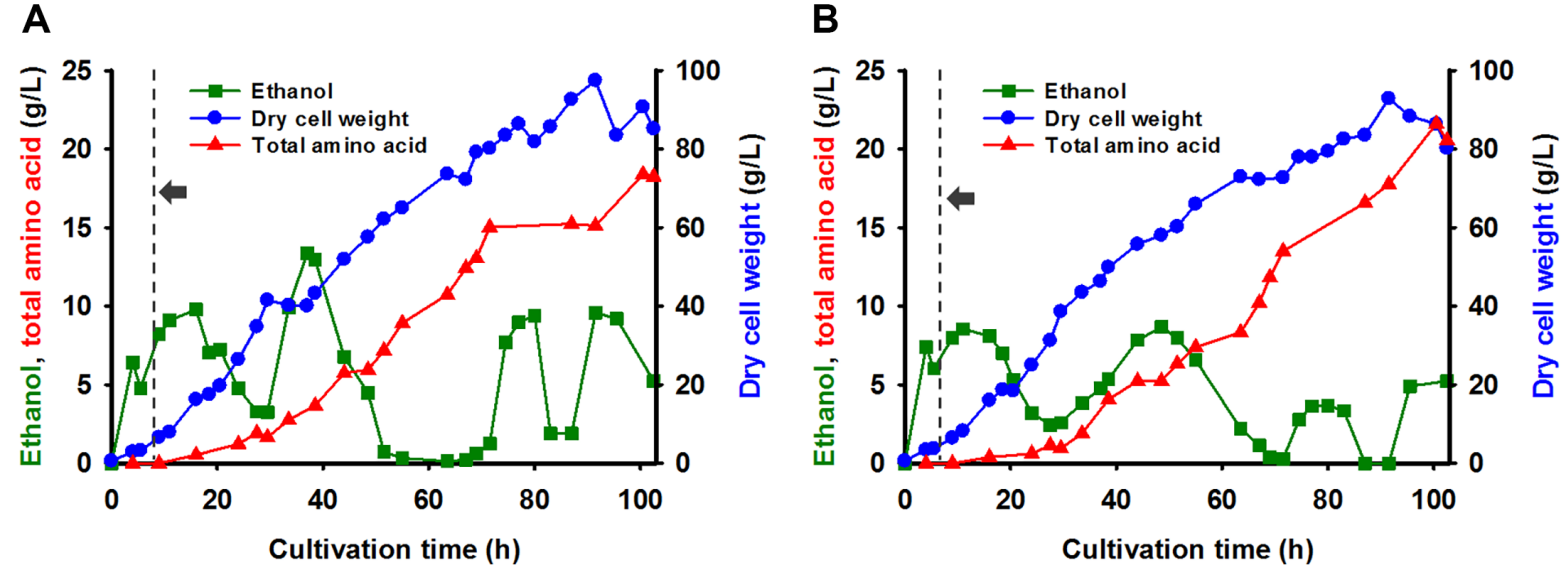
Glucose-limited fed-batch fermentation of the parental *Saccharomyces cerevisiae* KCCM 51811 (A) and its #126 mutant strain (B). Following the depletion of sugars present in a mixture of 4% molasses and 2% corn steep liquor (CSL), a feeding solution consisting of 50% molasses and 25% CSL was fed into the bioreactor (arrow). Throughout the cultivation period, the pH and temperature of the medium were maintained at 5.5 and 30°C, respectively. All values were measured in duplicate, and the results represent average values having less than 5% standard deviations.

To investigate the potential of the #126 mutant strain for industrial SCP production, fed-batch fermentations of *S. cerevisiae* KCCM 51811 and its #126 mutant strain were conducted using molasses and CSL in a 2.5-L scale bioreactor. Notably, SCP production was most efficient when molasses and CSL were added in a 2:1 ratio (Fig. 2); therefore, 4% (v/v) molasses and 2% (v/v) CSL were added during the initial batch fermentation stage. Following the depletion of sugars present in 4% (v/v) molasses, a feeding solution composed of 50% (v/v) molasses and 25%(v/v) CSL was fed, with rates ranging from 8.0 to 22.5 ml/h for the KCCM 51811 strain and 7.6 to 18.6 ml/h for the #126 mutant strain, to maximize both cell growth and SCP production. The growth patterns of the two *S. cerevisiae* strains were virtually identical, confirming that mutations in the #126 mutant strain had no discernable deleterious effects on *S. cerevisiae* growth (Fig. 6). Notably, the fed-batch culture of the #126 mutant strain yielded a maximum total amino acid concentration of 21.6 g/l, representing an 18% increase compared to the parental KCCM 51811 strain. However, the magnitude of the increase in SCP production observed in fed-batch fermentation was lower than that observed in batch fermentation. Further research efforts are currently underway to optimize fermentation conditions to improve the performance of the #126 mutant strain in fed-batch fermentation.

## Conclusion

This study aimed to develop an edible yeast strain with high protein content and concentration. To achieve this, initially, protein concentrations of 20 wild-type *S. cerevisiae* strains were compared. Consequently, the *S. cerevisiae* KCCM 51811 strain, which exhibited 78% higher protein concentration than the *S. cerevisiae* laboratory strain, D452-2, was selected as the background strain for SCP production. To further increase the protein content and concentration of the KCCM 51811 strain, random mutagenesis through UV irradiation, followed by Bradford protein assay-based screening, was performed. A two-stage screening process—initially at the 96-well plate level followed by a second-round screening at the flask level—facilitated the isolation of the #126 mutant strain, which exhibited improved protein content and concentration. Finally, conducting a high cell density fermentation using the #126 mutant strain resulted in a total amino acid concentration of 21.6 g/l. Given the prevalent concerns associated with the use of GMM in the food industry, the #126 mutant strain, developed using non-GMM strategies, holds the potential for efficient SCP production in this sector.

## Supplemental Materials

Supplementary data for this paper are available on-line only at http://jmb.or.kr.



## Figures and Tables

**Table 1:** Comparison of amino acid concentration (mg/l) in the parental *Saccharomyces cerevisiae* KCCM 51811 and its two mutant strains (#126 and #152).

Amino acid	KCCM 51811	#126 mutant	#152 mutant
Aspartic acid	418.6 ± 0.3^a^	567.0 ± 2.2^b^	529.6 ± 15.4^b^
Glutamic acid	456.2 ± 7.3^a^	614.1 ± 11.4^b^	573.9 ± 12.1^b^
Serine	266.7 ± 1.3^a^	354.5 ± 8.2^ab^	325.2 ± 17.9^b^
Histidine	103.9 ± 1.5^a^	144.6 ± 0.0^b^	127.0 ± 11.3^ab^
Glycine	255.6 ± 5.7^a^	343.9 ± 0.9^b^	318.4 ± 15.5^b^
Threonine	320.4 ± 5.2^a^	447.9 ± 23.4^b^	394.5 ± 17.8^ab^
Arginine	185.4 ± 27.4^a^	252.9 ± 36.8^a^	246.7 ± 52.0^a^
Alanine	292.0 ± 0.5^a^	388.6 ± 9.6^b^	352.5 ± 10.9^b^
Valine	303.4 ± 4.2^a^	412.5 ± 14.9^b^	375.3 ± 24.2^ab^
Phenylalanine	233.1 ± 4.1^a^	313.5 ± 10.1^b^	289.2 ± 11.5^b^
Tyrosine	133.6 ± 26.6^a^	186.2 ± 30.0^a^	177.6 ± 41.7^a^
Isoleucine	282.7 ± 3.5^a^	384.4 ± 9.9^b^	351.4 ± 21.2^ab^
Leucine	379.1 ± 2.7^a^	508.6 ± 8.8^b^	469.5 ± 23.1^b^
Lysine	363.4 ± 3.9^a^	481.5 ± 2.2^b^	460.2 ± 28.7^ab^
Proline	169.0 ± 10.4^a^	250.6 ± 7.9^b^	205.4 ± 13.8^ab^
Total	4163.0 ± 83.9^a^	5650.7 ± 13.6^b^	5196.3 ± 317.1^ab^

Total amino acid concentrations were determined after cultivation for 36 h in 100 ml YP50D medium (10 g/l yeast extract, 20 g/l Bacto Peptone, and 50 g/l glucose).

Results represent the mean ± standard deviation of *n* ≥ 2 experiments. Different letters represent significantly different means (Tukey HSD tests, *p* < 0.05).
