# Supplementary material for: Enhancing Protein Content in Wild-Type Saccharomyces cerevisiae via Random Mutagenesis and Optimized Fermentation Conditions
Source: J Microbiol Biotechnol. 2024 Jul 31;34(9):1912–8. doi: 10.4014/jmb.2405.05027 (PMC11485558; doi:10.4014/jmb.2405.05027)
Supplement: Supplementary file 1 [file jmb-34-9-1912-supple.pdf]

J. Microbiol. Biotechnol. 2024. 34(9): 1912-1918
https://doi.org/10.4014/jmb.2405.05027

Supplementary Tables and Figure

Table S1. A list of wild-type *Saccharomyces cerevisiae* strains used in this study.

Deposit number	Origin and/or characteristics
KACC 30068	Silage from Gyeonggi-do, Korea
KACC 47487	Yakju from Gyeonggi-do, Korea & protease activity
KACC 47715	Makgeolli from Gyeonggi-do, Korea & ethanol tolerance
KACC 48329	Makgeolli from Chungcheongbuk-do, Korea & high flocculation
KACC 94806	Makgeolli from Seoul, Korea & ethanol tolerance
KCCM 12241	Kaffir beer
KCCM 12498	Baker's yeast
KCCM 12500	Baker's yeast
KCCM 12632	Baker's yeast & high flocculation
KCCM 12650	Wine
KCCM 32016	Jamaican cane juice & molasses fermentation
KCCM 50460	Shao-Hsing wine
KCCM 50712	Molasses fermentation
KCCM 51286	Brewer's yeast
KCCM 51292	Wine from Gangwon-do, Korea & ethanol tolerance
KCCM 51811	Grape from Gyeongsangbuk-do, Korea
KCCM 90289	Nuruk
KCCM 90290	Nuruk
KCCM 90291	Nuruk
CCM 90292	Nuruk

Table S2. Comparison of total amino acid contents (%) in the parental *Saccharomyces cerevisiae* KCCM 51811 and its two mutant (#126 and #152) strains.

Amino acid	KCCM 51811	#126 mutant	#152 mutant
Aspartic acid	3.1 ± 0.1^{a}	4.5 ± 0.0^{c}	3.8 ± 0.1^{b}
Glutamic acid	3.4 ± 0.1^{a}	4.8 ± 0.1^{c}	4.1 ± 0.1^{b}
Serine	2.0 ± 0.0^{a}	2.8 ± 0.1^{b}	2.3 ± 0.1^{ab}
Histidine	0.8 ± 0.0^{a}	1.1 ± 0.0^{b}	0.9 ± 0.1^{ab}
Glycine	1.9 ± 0.1^{a}	2.7 ± 0.0^{b}	2.3 ± 0.1^{ab}
Threonine	2.4 ± 0.0^{a}	3.5 ± 0.2^{b}	2.8 ± 0.1^{ab}
Arginine	1.4 ± 0.2^{a}	2.0 ± 0.3^{a}	1.8 ± 0.4^{a}
Alanine	2.2 ± 0.1^{a}	3.1 ± 0.1^{b}	2.5 ± 0.1^{a}
Valine	2.3 ± 0.1^{a}	3.2 ± 0.1^{b}	2.7 ± 0.2^{ab}
Phenylalanine	1.7 ± 0.1^{a}	2.5 ± 0.1^{b}	2.1 ± 0.1^{ab}
Tyrosine	1.0 ± 0.2^{a}	1.5 ± 0.2^{a}	1.3 ± 0.3^{a}
Isoleucine	2.1 ± 0.1^{a}	3.0 ± 0.1^{b}	2.5 ± 0.1^{ab}
Leucine	2.8 ± 0.1^{a}	4.0 ± 0.1^{b}	3.4 ± 0.2^{ab}
Lysine	2.7 ± 0.0^{a}	3.8 ± 0.0^{b}	3.3 ± 0.2^{ab}
Proline	1.3 ± 0.1^{a}	2.0 ± 0.1^{b}	1.5 ± 0.1^{ab}
Total	31.0 ± 1.4^{a}	44.4 ± 0.0^{b}	37.4 ± 2.2^{ab}

Total amino acid concentrations were determined after cultivation for 36 h in 100 mL YP50D medium (10 g/L yeast extract, 20 g/L Bacto Peptone, and 50 g/L glucose).

Results represent the mean ± standard deviation of $n \geq 2$ experiments. Different letters represent significantly different means (Tukey HSD tests, $p < 0.05$).

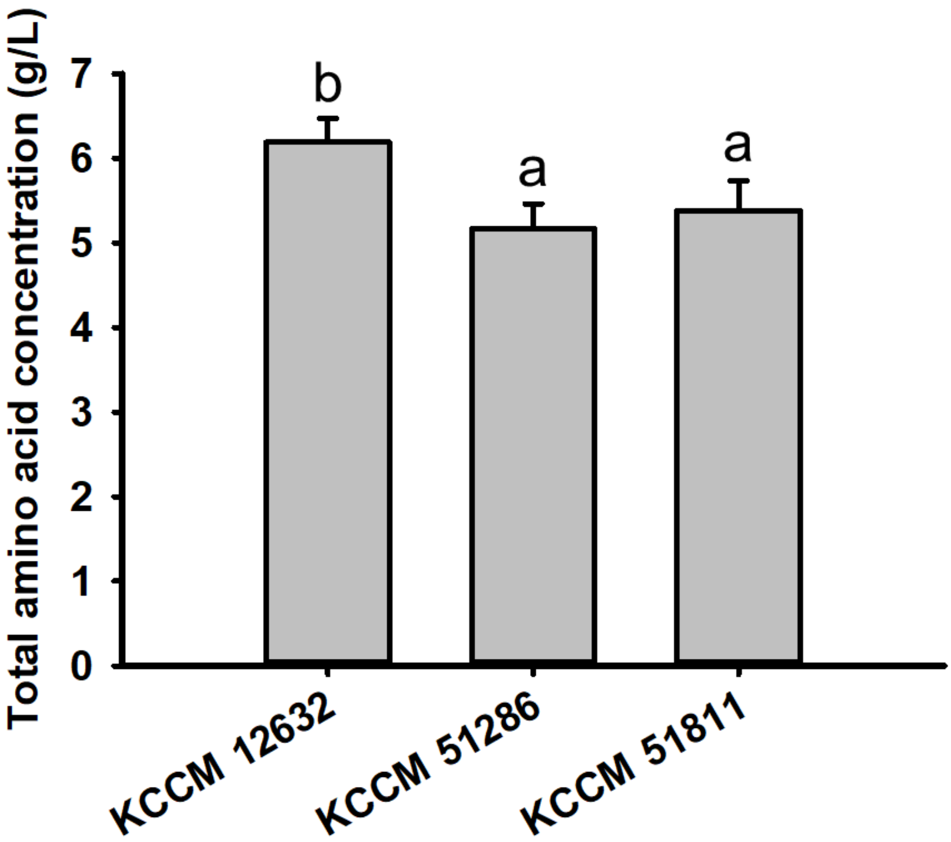

Fig. S1. Comparison of total amino acid concentrations in the three wild-type *Saccharomyces cerevisiae* strains (KCCM 12632, KCCM 51286, and KCCM 51811) with high protein concentration. Total amino acid concentrations were calculated through HPLC-based amino acid profiling. Results represent the mean of $n \geq 2$ experiments, with error bars indicating the standard deviation. Different letters represent significantly different means (Tukey HSD tests, $p < 0.05$).
